# Phenotypic Heterogeneity in Attachment of Marine Bacteria toward Antifouling Copolymers Unraveled by AFM

**DOI:** 10.3389/fmicb.2017.01399

**Published:** 2017-07-27

**Authors:** Sofiane El-Kirat-Chatel, Aurore Puymege, The H. Duong, Perrine Van Overtvelt, Christine Bressy, Lénaïk Belec, Yves F. Dufrêne, Maëlle Molmeret

**Affiliations:** ^1^CNRS and Université de Lorraine, Laboratoire de Chimie Physique et Microbiologie pour l’Environnement (LCPME), UMR 7564 Nancy, France; ^2^Institute of Life Sciences, Université catholique de Louvain Louvain-la-Neuve, Belgium; ^3^Laboratoire MAPIEM, EA4323, Université de Toulon La Garde, France; ^4^University of Science and Technology, The University of Danang Danang, Vietnam

**Keywords:** marine bacteria, adhesion, heterogeneity, AFM, copolymers surfaces

## Abstract

Up to recent years, bacterial adhesion has mostly been evaluated at the population level. Single cell level has improved in the past few years allowing a better comprehension of the implication of individual behaviors as compared to the one of a whole community. A new approach using atomic force microscopy (AFM) to measure adhesion forces between a live bacterium attached via a silica microbead to the AFM tipless cantilever and the surface has been recently developed. The objectives of this study is to examine the bacterial adhesion to a surface dedicated to ship hulls at the population and the cellular level to understand to what extent these two levels could be correlated. Adhesion of marine bacteria on inert surfaces are poorly studied in particular when substrata are dedicated to ship hulls. Studying these interactions in this context are worthwhile as they may involve different adhesion behaviors, taking place in salty conditions, using different surfaces than the ones usually utilized in the literacy. FRC (fouling release coatings)–SPC (self-polishing coatings) hybrids antifouling coatings have been used as substrata and are of particular interest for designing environmentally friendly surfaces, combining progressive surface erosion and low adhesion properties. In this study, a hybrid coating has been synthetized and used to study the adhesion of three marine bacteria, displaying different surface characteristics, using microplate assays associated with confocal scanning laser microscopy (CSLM) and AFM. This study shows that the bacterial strain that appeared to have the weakest adhesion and biofilm formation abilities when evaluated at the population level using microplates assays and CSLM, displayed stronger adhesion forces on the same surfaces at the single cell level using AFM. In addition, one of the strains tested which presented a strong ability to adhere and to form biofilm at the population level, displayed a heterogeneous phenotypic behavior at the single cell level. Therefore, these results suggest that the evaluation of adhesion at the population level cannot always be correlated with adhesion forces measured individually by AFM and that some bacteria are prone to phenotypic heterogeneity among their population.

## Introduction

Little is known on adhesion of marine bacteria on surfaces in particular when they are dedicated to ship hulls. The comprehension of bacterial adhesion on these surfaces should help finding potential environmentally less toxic anti-adhesion or anti-fouling strategies. The specific intrinsic nature of marine bacteria, that are poorly studied and characterized, may modify the type of interactions that can be observed between a cell and its surface, particularly when the interactions take place in marine conditions, making them worthwhile studying. Overall, molecular or cellular mechanisms of bacterial adhesion have been in fact mostly evaluated at the population level but very rarely at the single cell level. Indeed, very few information are factually available on individual behaviors of bacteria regarding adhesion. Due to the development of new single cell level approaches, individual cells can be studied with the purpose of understanding how a single cell behaves as compared to its population of origin and if bacterial cells behave all similarly within a supposedly clonal population, after synchronization in growth culture, or if important behavioral differences exist between them. Recently, the idea that bacterial population could be composed of heterogeneous individuals has emerged, even when coming from a single cell or a group of clonal or genetically identical individuals (Grote et al., 2015; Martins and Locke, 2015). Differential gene expression could explain these phenotypic fluctuations. Some bacterial strains are also more prone to allelic variations than others (Davis and Isberg, 2016). In adhesion studies, atomic force microscopy (AFM) approaches have been used to study adhesion forces at the single cell level between a cell and a surface. They have been improved during the past few years, making it possible to study these interactions with alive bacteria (Kang and Elimelech, 2009; Loskill et al., 2012; Beaussart et al., 2013, 2014; El-Kirat-Chatel et al., 2014a). Indeed, a new approach using a silica microbead fixed on the tipless cantilever allows the attachment of a single cell that can stay alive during the time of the measurement. These approaches have been proven very useful to decipher adhesion of bacteria such as *Staphylococcus aureus, Escherichia coli* toward glass and functionalized surfaces.

In the marine context, all artificial surfaces immersed in seawater are subjected to the accumulation of marine organisms such as microorganisms and macrofoulers, known as marine biofouling. Current antifouling strategies rely on the wide use of self-polishing coatings (SPC), which release toxic biocides with a constant rate controlled by the coating erosion process (Yebra et al., 2004). The erosion of the coating is achieved through the hydrolysis of the polymeric binder in seawater making the polymer water-soluble. Fouling release coatings (FRC) represent a second type of antifouling coatings, which are able to release organisms settled on the surface while boats are navigating (Lejars et al., 2012). Their efficacy relies on hydrophobicity, low surface energy and low elastic modulus of its poly(dimethylsiloxane) (PDMS) cross-linked matrix, which decreases the adhesion strength of marine organisms and enhance their removal. Despite, the clear environmentally friendly advantage of this antifouling solution, FRCs are inefficient when vessels are docked. During navigation, the coating is able to release the macrofouling but retains a microfouling film (composed mainly of bacteria and diatoms), which is still responsible for 10% of drag resistance (Schultz, 2007). An attractive option in developing such coatings is the synthesis of new polymers which are both hydrolyzable and hydrophobic/low-surface energy materials. Poly(dimethylsiloxane) blocks could be inserted in silylated-based polymers to provide access to a wide variety of materials with tunable hydrophobicity, water resistance and mechanical properties. Bressy and Margaillan (2009), Bressy et al. (2010, 2014), Lejars et al. (2014) have synthesized tri-alkylsilylester-based statistical copolymers by conventional radical polymerization and several diblock copolymers using the reversible addition-fragmentation chain transfer (RAFT) polymerization for developing erodible binders for marine antifouling coatings. Hybrid copolymers with PDMS blocks or side-chains and silylated side groups have been reported to exhibit surface erosion and hydrophobic surfaces depending on the relative content of the two components (Lejars et al., 2013). These hybrid surfaces displaying SPC and FRC properties have been characterized including for their antifouling efficacy (Duong et al., 2014, 2015).

In this study, three strains isolated from the Mediterranean sea, presenting different phenotypical traits, have been used to evaluate their ability to adhere on a new antifouling coating dedicated to ship hulls at the population and the cellular level (Brian-Jaisson et al., 2014). TC5 belonging to the *Polaribacter* genus, a non-motile marine bacteria, is the most hydrophobic of the three strains according to Microbial Adhesion to Solvents (MATS) assays and has a poor ability to form biofilm on polystyrene when studied in microplates (Brian-Jaisson et al., 2014). TC10 and TC11 are two different strains of *Shewanella*, which are overall more hydrophilic and are motile. TC11 is able of a stronger adhesion and a faster capacity to form a biofilm on polystyrene while for TC10, it takes more time to form its biofilm. In this context, adhesion have been tested on an hybrid block copolymer SPC-FRC coating called MC3MB6 [PDMS-*b*-p(SiMA-*stat*-BMA)]. The results have been compared to its SPC block alone called MB6 (SiMA-*stat*-BMA). In contrast with conventional SPC, MB6 has no biocide but retaining the ability of self-hydrolysis. Both surfaces have been synthesized and their properties characterized similarly as previously done (Duong et al., 2014, 2015). The adhesion assays of these three marine strains on the hybrid coatings have been performed through a microplate assay associated with CLSM and AFM, in order to verify if the adhesion forces measured at the single cell level could be correlated with the evaluation of the population adhesion. Bacterial adhesion has been very rarely evaluated at the same time, at the population and the cellular level to understand to what extent these two levels could be correlated for each of the strain.

## Materials and Methods

### Substrates

Two copolymers based on *Tert*-butyldimethylsilyl methacrylate (SiMA) were synthesized as previously reported (Duong et al., 2014). Butyl methacrylate was used as co-monomer of SiMA to prepare films without cracking (**Table 1**). Each copolymer was dissolved in toluene, at a 40–50 wt% solid content, and applied on abraded poly(vinyl chloride) (PVC) substrates with a bar-coater resulting in about 100 μm dried thickness coatings. The surfaces of the samples for the contact angle measurement and for AFM measurements were 25 mm × 45 mm and 10 mm × 10 mm, respectively. The coated plates were left to dry in the open air for 15 days.

**Table 1:** Characteristics of diblock and statistical copolymers prepared by reversible addition-fragmentation chain transfer (RAFT) polymerization of SiMA and BMA from PDMS-macro RAFT agent at 70°C in toluene.

Polymer	%_mol_ (DMS/SiMA/BMA)	%_mass_ (DMS/SiMA/BMA)	% *vol_PDMS_*	Mn (g.mol^-1^)^∗^	*Ð*^∗^
MC3MB6	31/10/59	18/16/66	19	59,700	1.1
MB6	0/14/86	0/18/82	0	49,500	1.1

*^∗^Assessed by triple detection size exclusion chromatography (TD-SEC).*

### Characterization Methods

The number-average molar mass (*M*_n_) and dispersity (*Ð*) of polymers were determined by triple detection size exclusion chromatography (TD-SEC). Analyses were performed on a Viscotek apparatus, composed of a GPC Max (comprising a degasser, a pump and an autosampler) with a TDA-302 (RI refractive index detector, right and low angle light scattering detector at 670 nm and viscometer) and an UV detector (λ = 298 nm). The following columns were used: a Viscotek HHR-H precolumn and two Viscotek ViscoGel GMHHR-H columns. THF was used as the eluent with a flow rate of 1.0 mL min^-1^ at 30°C. For each precipitated polymer, the refractive index increment (d*n/*d*c*) was determined using the OmniSec software, from a solution of known concentration (ca. 10 mg mL^-1^) filtered through a 0.2 mm PTFE filter.

Differential scanning calorimetric (DSC) measurements were performed on a DSC Q10 apparatus from TA Instruments calibrated with indium. Polymer samples weighing 15–20 mg were run at equal heating and cooling rates, 10°C min^-1^, under a constant stream of nitrogen. The MC3MB6 sample was first scanned from room temperature to 100°C [PDMS-*block*-P(SiMA-*stat*-BMA)]. The sample was then cooled to -165°C. This temperature was held for 5 min to allow the system to attain thermal equilibrium before the second heating scan. The first heating ramp of each sample was discarded for this work. The glass transition temperature (*T*_g_) values were determined as the midpoint between the onset and the end of a step transition using the TA Instruments Universal Analysis 2000 software.

Static contact angle measurements were carried out at room temperature using a sessile drop method with a DIGIDROP contact angle meter from GBX Instruments. Two test liquids: deionized water and diiodomethane (Sigma–Aldrich) were used. The liquid drop volume was 1 and 0.5 μL for water and diiodomethane, respectively. A picture of the liquid drop on the surface was taken 4 s after its formation for contact angle measurement. The reported contact angles were an average of five individual measurements in different regions of the same coating (±σ). Surface free energies of the coatings (*γ*_s_) and their dispersive (*γ*_s_^D^) and polar components (*γ*_s_^P^) were calculated using the Owens–Wendt method. Dynamic contact angle measurements were carried out under ambient conditions by using the dynamic sessile drop technique. A water drop with a volume of around 1 μL is growing on a syringe tip and picked up by the surface. The syringe tip never leaves the liquid drop. The water was inflated and sucked up from the surface and the advancing and receding angles were obtained.

### AFM Characterization of the Surface

Atomic force microscopy measurements were performed on a Nanoscope V controller equipped with a Multimode V Atomic Force Microscope, with a 8610 JVLR type scanner. Tapping mode cantilever probes (RTESP model from BRUKER) were used to show the topography of the supported polymer films and to evaluate their Young modulus values. The system sensitivity and cantilever spring constant kc are successively determined from force measurements on a rigid sample and from the thermal tune method (Butt et al., 2005), implemented in Bruker Nanoscope (V7.3) software. The topography was initially scanned in tapping mode with a cantilever spring constant around 48 N/m and a resonance frequency of ∼ 380 kHz. AFM force curves were performed with maximum forces lower than 1.5 μN (The slope of the force–displacement approach curve in the linear elastic range gives an apparent stiffness keff which is directly linked to the sample stiffness ks knowing the cantilever stiffness (Butt et al., 2005). In the case of a perfectly elastic tip with a spherical end and a homogeneous sample, with no adhesive effects, the Hertz model can give an estimation of Young’s modulus from ks measurement (Butt et al., 2005; Belec et al., 2015). The slope was calculated on the approach curves (between 400 and 450 nm of deflection). The standard deviation is calculated on seven measurements.

### Microorganisms and Growth Conditions

Bacterial strains used in this study (TC for Toulon Collection) are listed in Supplementary Table S1. They were isolated from biofilms formed on inert surfaces immersed in the Mediterranean Sea (bay of Toulon, France, 43°06′23″N-5°57′17″E) (Camps et al., 2011; Briand et al., 2012). TC strains were grown in Vaatanen nine salt solution (VNSS) at 20°C in a rotary shaker (120 rpm) (Mardén et al., 1985) up to post-exponential phase prior to analysis.

### Adhesion Assays on Polystyrene

Post-exponential phase grown cells were centrifuged and resuspended in artificial sea water (ASW). Then 200 μL of cells were inoculated at OD_600 nm_ 0.3 in triplicate in black microplates (sterile black PS; Nunc, Fisher Scientific, Illkirch, France). After 24 h of incubation at 20°C, the non-adhered bacteria were eliminated by three successive washes (36g.L^-1^ sterile NaCl solution). The adhered bacteria were stained by both Syto 61 Red and Syto 9 Green fluorescent markers (5 μM) targeting bacterial DNA (Life technology). After 10 min, the excess stain was eliminated by one wash. Fluorescence intensity (FI) was measured using an Infinite 200 microplate fluorescence reader (Tecan, Lyon, France). A fluorescent intensity was calculated per well: Fluorescent intensity (FI) = FI average assay/FI average negative control. Three independent assays were done for each strain tested. Same results were found with both stains (data not shown).

### Adhesion Assays on Copolymers

Adhesion assay on copolymers were performed as described for the adhesion assays on polystyrene excepted for the following points. PVC coverslips of 13 mm of diameter were coated with the MB6 and MC3MB6 polymers. Each copolymer previously dissolved in toluene, at a 40–50 wt% solid content, were applied on PVC coverslips with a bar-coater resulting in about 100 μm dried thickness coatings. Coverslips were inserted in 24 well microplates (sterile transparent PS; VWR) and sterilized 15 min with UV. Post-exponential bacterial strains were resuspended in ASW and inoculated at OD_600 nm_ 1 in the microplates. After 24 h of incubation at 20°C strains were labeled with 5 μM of Syto 9 Green fluorescent nucleic acid stain (Life technology). After 10 min, the excess stain was eliminated by three washes. FI was measured using an Infinite 200 microplate fluorescence reader (Tecan, Lyon, France). A fluorescent intensity was calculated per well: FI = FI average assay/FI average negative control. Three independent assays were done for each strain tested.

### CSLM Observation

The same coverslips were used for the observation of the bacteria on the surfaces using confocal scanning laser microscopy (CSLM) Zeiss LSM 510. Briefly, the coated coverslips were glued onto a glass slide and covered with prolong antifade (Life technology) and a new glass coverslips. After 48 h drying, the samples were stored at 4°C until use for CSLM observation.

### Statistical Analysis

GraphPad Prism 5 (GraphPad Software, San Diego, CA, United States) was used for statistical analysis of the adhesion assays. Data were analyzed using one-way ANOVA and treatment effects were separated using Turkey’s multiple comparison *post hoc* tests. Statistical significance was accepted at p <0.05.

### Atomic Force Microscopy Imaging

Atomic force microscopy contact mode images were obtained in air, at room temperature, using a Nanoscope VIII Multimode AFM (Nano Surfaces Business, Bruker Corporation, Santa Barbara, CA, United States), MSCT cantilevers with a nominal spring constant of ∼0.01 N/m (calculated with the thermal noise method), and a scanning rate of 2 Hz. One hundred μl of cell suspension from post-exponential growth phase was put in contact with freshly cleaved mica supports mounted on steel pucks. The samples were incubated for 2 h at 30°C, gently rinsed in three successive baths of ultrapure water (Elga, purelab), and allowed to dry at 30°C overnight.

### Cell Probes

For single-bacterial cell force spectroscopy, cell probes were prepared using a recently developed protocol that combines colloidal probe cantilevers and bioinspired polydopamine wet adhesives (Beaussart et al., 2013). Briefly, silica microspheres (6.1 μm diameter, bangs laboratories) were attached on triangular shaped tipless cantilevers (NP-O10, Microlevers, Bruker Corporation) using UV-curable glue (NOA 63, Norland Edmund Optics). The cantilevers were then immersed for 1 h in a 10 mM Tris Buffer solution (pH 8.5) containing 4 mg ml^-1^ dopamine hydrochloride (99%, Sigma), and dried with N_2_ flow. Single bacteria were then attached onto polydopamine-coated colloidal probes using a Bioscope Catalyst AFM (Bruker corporation, Santa Barbara, CA, United States). To this end, 2 μl of a cell suspension were added to 4 ml of ASW solution (pH 8, Sea salts, Sigma) in a glass Petri dish containing MB6 and MC3MB6 substrates. A single probe was brought into contact with an isolated cell for 3 min, and the obtained cell probe was then transferred over a solid substrate for further force measurements. Viability of the attached bacteria was checked using a Live-dead *Bac*light viability kit (Invitrogen, kit L7012) following the manufacturer instructions.

### Single-Cell Force Spectroscopy Measurements

Single-cell force spectroscopy (SCFS) measurements were performed at room temperature (20°C) in ASW solution pH 8 and using a Bioscope Catalyst AFM (Bruker AXS Corporation, Santa Barbara, CA, United States). The nominal spring constant of the colloidal probe cantilever was ∼0.06 N m^-1^, as determined by the thermal noise method. Multiple force-distance curves were recorded on various spots of MB6 and MC3MB6 substrates using a maximum applied force of 250 pN, a contact time of 100 ms or 1 s, and constant approach and retraction speeds of 1000 nm s^-1^. For each condition, the interaction forces of three bacterial cells from independent cultures were measured and *n* > 400 force curves were recorded for each bacteria.

## Results and Discussion

### Polymers Synthesis and Characterization of Copolymer Surfaces

**FIGURE 1:**
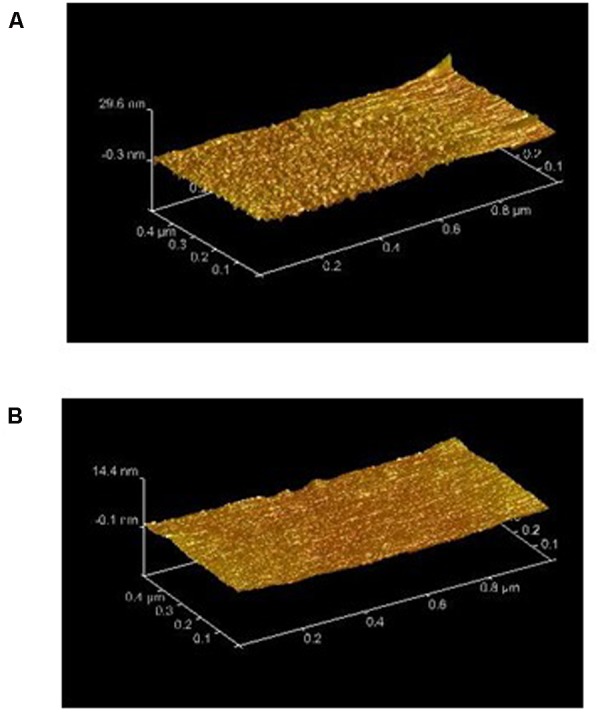
Height atomic force microscopy (AFM) images of **(A)** MB6 and **(B)** MC3MB6. Rms is 2.3 ± 0.10 and 5.4 ± 1 for MC3MB6 and MB6, respectively, with Ra of 8.6 ± 0.6 and 14 ± 7, respectively.

Well-defined diblock copolymers combining a tert-butyl dimethylsilyl methacrylate (SiMA)-based block, as hydrolyzable “SPC-type” monomer, with a poly(dimethylsiloxane) (PDMS) block, as hydrophobic, “FRC-type” monomer have been investigated. The synthesis of the PDMS-*b*-p(SiMA-*stat*-BMA) block copolymer called MC3MB6 was achieved from copolymerizations of *tert*-butyldimethylsilyl methacrylate (SiMA) and butyl methacrylate on PDMS macro-RAFT agents. The methodology relies on the synthesis of PDMS monofunctional chain transfer agents easily available in one synthetic step from commercially available hydroxylated PDMSs (Duong et al., 2014). A statistical copolymer P(SiMA-*stat*-BMA) called MB6, with a composition similar to the second block of MC3MB6, has also been prepared (**Table 1**). As these copolymers might be used in marine environment as coatings, their ability to form films without cracking is required. Good film properties have been displayed for MB6 and MC3MB6 due to their low glass transition temperature of 45–46°C corresponding to P(SiMA-*stat*-BMA) block. In the case of MC3MB6, the *T*_g_ of the PDMS block (from -127 to -124°C) was not visible because of a low amount of DMS monomer units within the copolymer (Duong et al., 2014). Surface properties including wetting properties and smoothness have been investigated. **Table 2** shows that the water contact angle values increased and the polar component of the surface free energy (γ_s_^P^) decreased when the PDMS block was added within the copolymer. Taken together these results show that MC3MB6 is more hydrophobic than its MB6 counterpart which could suggest according to the literature a close packing of the pendant methyl groups of the flexible siloxane chain at the film/air interface (Lejars et al., 2012). Tapping-mode AFM analysis shows the topography of the PDMS-based films to be smoother than the MB6 PDMS-free coating (**Figure 1** and Supplementary Figure S1). In addition, the flexibility of the PDMS block coming from its low *T*_g_ value and the flexibility of the methacrylic block coming from the presence of BMA monomer units provided soft samples. A lower Young’s modulus value and a higher indentation were found for the PDMS-based sample (**Table 3**). Taken together these results show that MC3MB6 surface is softer than MB6 one.

**Table 2:** Wetting properties of the coating surfaces.

Polymer	Contact angle (°)				Surface energies (mJ.m^-2^)^∗^		
	𝜃_H2O_	σ	𝜃_CH2I2_	σ	γ*_s_*	γ_s_^D^	γ_s_^P^
MC3MB6	101.9	1	71.5	0.6	22	20.9	1.1
MB6	91.3	0.5	64.3	4.6	26.7	23.3	3.3

*^∗^Using Owens–Wendt’s method (Owen and Wendt, 1969).*

**Table 3:** Young’s modulus at the coating surfaces measured by AFM.

Polymer	*E* (MPa)	Indentation (nm)
MC3MB6	73 ± 4	207 ± 4
MB6	88 ± 7	145 ± 5

**FIGURE 2:**
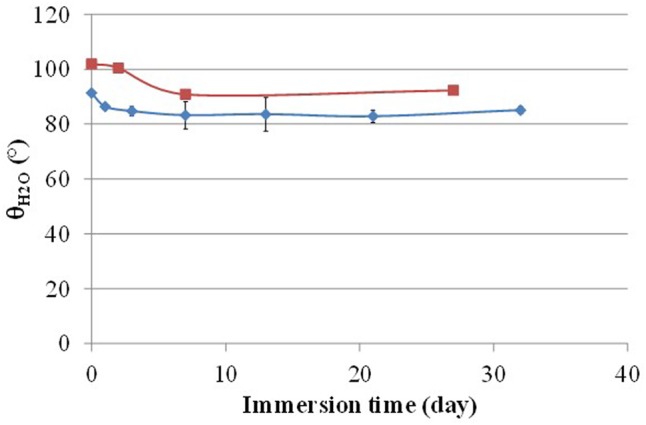
Evolution of the static water contact angle of MC3MB6 (■) and (♦) MB6-based coatings with ASW immersion time.

When immersing these silylester-based polymers in artificial seawater, the hydrophilic character of the two coating surfaces increased with time as the water contact angle 𝜃_H2O_ decreases with immersion time (**Figure 2**). This result is in agreement with the well-known hydrolysis reaction of the hydrophobic silyl ester groups of SiMA units into hydrophilic sodium carboxylate groups in artificial seawater (Bressy and Margaillan, 2009). Nevertheless, the surface of the PDMS-based coating MC3MB6 remains more hydrophobic than the MB6 one.

### Adhesion Tests of Marine Bacteria on Polystyrene

**FIGURE 3:**
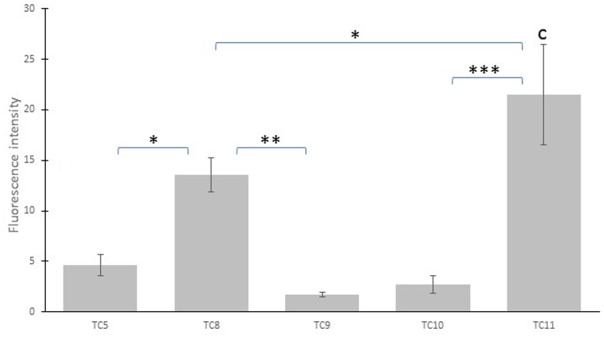
Evaluation of the adhesion of five marine bacterial strains on polystyrene. After 24 h of incubation at 20°C, bacteria were stained by Syto 9 Green and fluorescent intensity was measured as a representation of bacterial adhesion. Bars represent the standard deviation obtained from three independent measures. TC5 is a *Polaribacter* sp. strain, TC8 is a *Pseudoalteromonas lipolytica* strain, TC9, TC10, and TC11 are three strains of *Shewanella*. Bars represent the standard deviation obtained from three independent measures. Statistical significance was accepted at *p* < 0.05. ^∗^*p* < 0.05, ^∗∗^*p* < 0.01, ^∗∗∗^*p* < 0.001.

Biofilm formation has been previously evaluated in different rich marine media for a number of marine bacteria isolated from the Mediterranean sea (Camps et al., 2011; Brian-Jaisson et al., 2014). Five of these marine bacterial strains (Supplementary Table S1), which were all isolated from biofilms formed on immersed supports in the bay of Toulon (France), were analyzed for their adhesion ability in ASW on polystyrene (**Figure 3**). Three of them (TC9-TC10 and TC11) belong to *Shewanella* genus. TC5 and TC8 belong, respectively, to *Polaribacter* and *Pseudoalteromonas* genus (Supplementary Table S1). All strains except TC5, were able to form a biofilm in laboratory conditions (Brian-Jaisson et al., 2014). In this study, strains exhibited different adhesion patterns on polystyrene after 24 h (**Figure 3**) in a poor medium, ASW. Bacterial adhesion of TC11 was the strongest. TC8 adhered to polystyrene but fluorescence intensity was 1.6 times less than for TC11. Adhesion on polystyrene was weak for TC5 and very weak for the TC10 and TC9 strains. Three profiles based on adhesion on polystyrene and biofilm formation pattern can be identified: (i) a weak adhesion profile in ASW with an incapacity to form biofilm in rich media (Brian-Jaisson et al., 2014) for TC5; (ii) a strong adhesion on polystyrene with a strong ability to form biofilm in rich media for TC8 and TC11; (iii) a weaker ability to adhere on polystyrene in ASW and a slower capacity to form a biofilm in rich media for TC9 and TC10, corresponding thus to an intermediary phenotypic between the two first groups. For the following approaches, we therefore chose to work with one strain of each group. TC5 belonging to the *Polaribacter* genus, a non-motile marine bacteria, is the most hydrophobic of the three strains according to Microbial Adhesion to Solvents (MATS) assays, has a weak adhesion profile and has a poor ability to form biofilm (Brian-Jaisson et al., 2014). TC10 and TC11 are two different strains of *Shewanella*, which are overall more hydrophilic and are motile. TC11 is able of a stronger adhesion and a faster capacity to form a biofilm on polystyrene while for TC10, it takes more time to form its biofilm (Brian-Jaisson et al., 2014).

### AFM Imaging to Unravel Morphological Features of Bacteria Species

**FIGURE 4:**
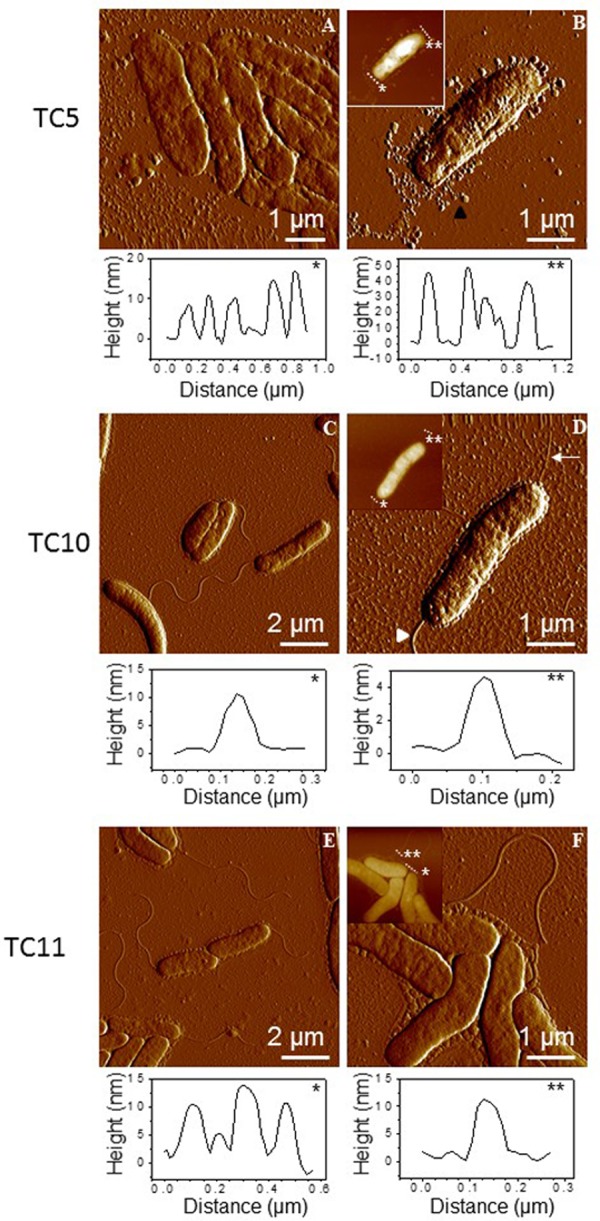
Imaging bacterial surface in air. AFM images of TC5 **(A,B)**, TC10 **(C,D)**, and TC11 **(E,F)** were performed in air using contact mode. AFM deflection and height (insets) images of post-exponentially growing cells that were directly deposited on mica and dried prior analysis. Vertical cross sections taken in the height images (asterisks indicate the correspondence with dashed lines) are also shown to emphasize sizes of cellular structures.

We used AFM contact mode imaging in air to visualize the general cell topography of TC5, TC10, and TC11. For all strains, bacteria were small rod-shaped, which agree well with observations performed previously (Brian-Jaisson et al., 2014). TC5 and TC10 were about 3.4 μm long. TC11 seemed to be smaller and was about 2.6 μm long (**Figure 4**).

Pili and flagella are major contributors to mobility, adhesion and biofilm formation (Mattick, 2002; Telford et al., 2006; Pelicic, 2008; Belas, 2014; Laverty et al., 2014). As suspected flagella were clearly present on the surface of TC10 and TC11, while they were not seen for TC5 (**Figure 4**), previously described as non-motile (Brian-Jaisson et al., 2014). Furthermore, in few images of TC10, we observed a smaller and thinner structure, which could be pili, on the surface of this strain (**Figure 4D**, white thin arrow). Overall, pili were more difficult to observe than flagella. It is possible that pili were broken during the preparation of the cells for the AFM observation. Despite the presence of small residues particles most likely coming from the culture medium, spherical particles, which surround TC5 strain seem to be of different nature (**Figure 4B**, black triangle). We hypothesized that this strain produces outer membrane vesicles (OMV). A wide variety of Gram-negative bacteria secrete OMV including marine bacteria such as *Prochlorococcus* or *Shewanella vesiculosa* (Beveridge, 1999; Perez-Cruz et al., 2013; Biller et al., 2014). OMV are implicated in many functions such as bacterial survival, pathogenicity, enzyme delivery and biofilm formation (Beveridge, 1999; Schooling and Beveridge, 2006; Lee et al., 2008; Yonezawa et al., 2009; Baumgarten et al., 2012; van Hoek, 2013; Altindis et al., 2014; Murphy et al., 2014; Orench-Rivera and Kuehn, 2016). Taken together, these observations show that the *Polaribacter* TC5 strain presents different features from the 2 *Shewanella* strains as it has no flagella and seems to present OMV at its surface.

### Evaluation of Adhesion on Copolymers at the Population Level

**FIGURE 5:**
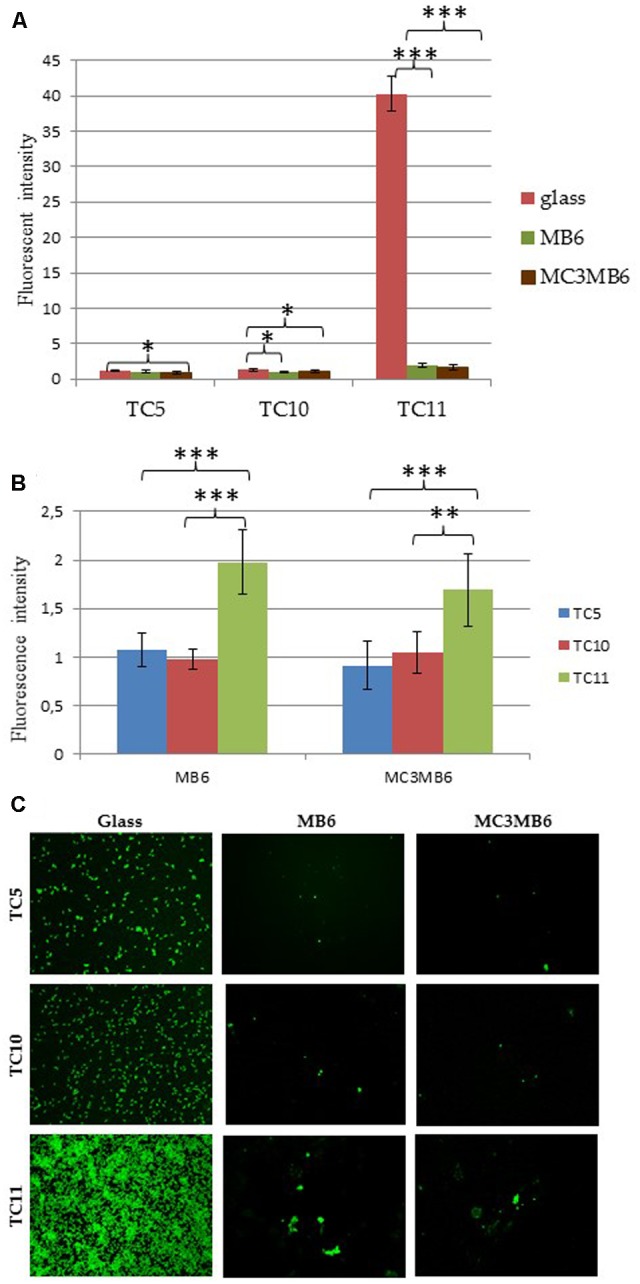
Adhesion of TC5, TC10, and TC11 on MB6 and MC3MB6 surfaces. Adhesion was first measured at the population level in 24 well plates. **(A,B)** Are different statistical analysis displays of the same measurement. After 24 h of incubation at 20°C, bacteria were stained by Syto 9 green fluorescent nucleic and fluorescent intensity was measured using a TECAN microplate reader as a representation of bacterial adhesion. Bars represent the standard deviation obtained from three independent measures. Statistical significance was accepted at *p* < 0.05. ^∗^*p* < 0.05, ^∗∗^*p* < 0.01, ^∗∗∗^*p* < 0.001. **(C)** Show the CSLM visualization of adhered bacteria on the surfaces. The same coverslips were used in the microplate assay and in CSLM. Glass coverslips were used as a control.

In order to evaluate bacterial adhesion on the hybrid MC3MB6 and its control MB6, these polymers were coated onto round PVC coverslips (as they did not stick well on glass) and inserted in 24 well plates. Glass coverslips, widely used in fluorescence or CLSM microscopy experiments, served as a reference. Bacteria were then left to seed onto the surface for 24 h, washed off to remove non-adherent bacteria and then stained using the fluorescent marker Syto9. Because some polymers can present an autofluorescence, a direct observation of the same samples was performed using CLSM. This double approach is rarely undergone when such coatings are used. **Figures 5A,B** show that for each coating tested, TC11 was the strain displaying stronger adhesive properties, in particular with glass alone. TC11 adhered 2 and 1.5 times more than TC5 or TC10 on MB6 or MC3MB6, respectively (**Figure 5B**). All three strains adhered better on glass coverslips than on the polymers (**Figures 5A,C**), with no significant difference in adhesion between MB6 and MC3MB6 (**Figure 5A**). These results have been corroborated with the CSLM observation as very few bacteria can be seen on either MC3MB6 or MB6 surfaces in sharp contrast with the glass surface (**Figure 5C**). Taken together, these results suggest that copolymers MB6 and MC3MB6 prevent bacterial adhesion. While the adhesion was overall very weak, TC11 appears to adhere slightly better on these two surfaces. MB6 alone, composed of SiMA-*stat*-BMA with self-hydrolysis properties (with no biocide) is self-sufficient for the inhibition of bacterial adhesion in these conditions. The hydrophobic PDMS blocks, do not add in efficacy whichever bacteria studied despite their different hydrophilic surface properties (Brian-Jaisson et al., 2014). As previously described, hydrophobic/hydrophilic interactions can be easily overcome by the presence of extracellular appendages and covalent bindings, in particular in the stage of the “irreversible adhesion” (Garrett et al., 2008). Bacteria can adapt to their environment, i.e., the presence of a surface, very rapidly by, temporarily and in coordinated manner, specifically expressing numbers of proteins anchored in the membrane or being part of the extracellular appendages that can modify and overcome such interactions.

### Single-Cell Adhesion Force Analysis

**FIGURE 6:**
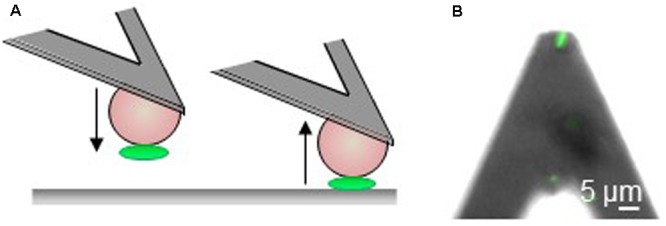
The use of a microbead for single-cell force spectroscopy analysis. **(A)** Principle of single cell force spectroscopy with tip less cantilevers modified with colloidal beads and coated with polydopamine to attach a single bacteria (green) and probe it toward surfaces. **(B)** Optical microscope image of a single bacterium attached to the colloidal cantilever probes documenting that the cell is properly located and alive (green fluorescence).

The results of the previous experiments reflect the behavior of the bacteria at the population level. In order to understand how each bacterium behaves on these surfaces at the single cell level, AFM was used in SCFS mode (Helenius et al., 2008; Muller et al., 2009) to quantify the adhesive properties of individual TC5, TC10, and TC11 bacterial cells toward surfaces MB6 and MC3MB6 (Beaussart et al., 2013). Briefly, a colloidal probe cantilever coated with polydopamine bioadhesive was used to pick up single cells without altering their viability (assessed using the Live-dead *Bac*light viability kit) and to measure force-distance curves between the bacterium and the surfaces MB6 and MC3MB6 (**Figure 6**). The three bacteria tested stayed alive during the course of the experiment.

**FIGURE 7:**
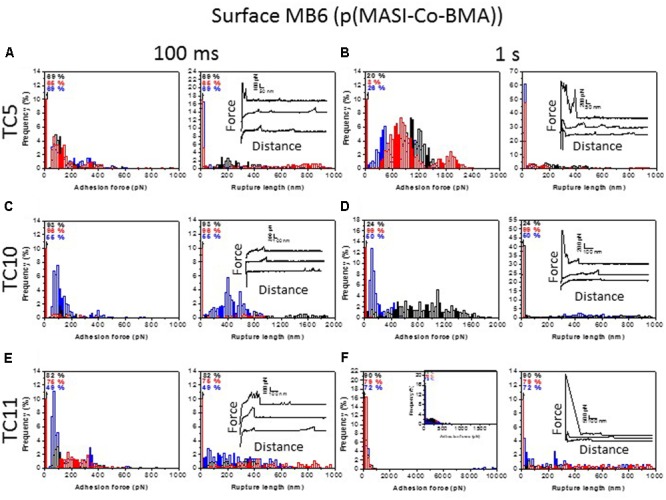
Single-cell force spectroscopy analysis on surface MB6. Adhesion force (left) and rupture length histograms with representative retraction force profiles (right) obtained by recording multiple force-distance curves between single TC5 **(A,B)**, TC10 **(C,D)**, or TC11 **(E,F)** bacteria and surface MB6 at short (100 ms, **A,C,E**) or prolonged (1 s, **B,D,F**) contact times. Black, red, and blue colors represent results from three cells from independent cultures (*n* > 400 force-distance curves for each cell).

We first used SCFS to investigate the adhesion force of single cells toward MB6 surfaces and the effect of contact time between cells and substrates. **Figure 7** shows the adhesion force and rupture length histograms, together with representative force curves, obtained between TC5 (**Figures 7A,B**), TC10 (**Figures 7C,D**), and TC11 (**Figures 7E,F**) cells and MB6 surfaces at short (**Figures 7A,C,E**) and prolonged (**Figures 7B,D,F**) contact times. Consecutive force curves were recorded on different spots of the substrate and no changes were observed regarding the general features of the curves, indicating the cells were not damaged and cell surface properties were not altered by force measurements. Cell from independent cultures were analyzed and generally yielded similar behavior although sometimes one cell showed differences (**Figures 7C,D,F**) that we attribute to heterogeneity of the bacterial population. This phenotypic heterogeneity was less pronounced for TC5, whereas it was more obvious for TC11 whether at a short or long contact time. At short contact time, the adhesion frequency of TC5 cells on MB6 surfaces was ∼30–35% with adhesive force curves presenting force of 50–400 pN and rupture distances of 100–900 nm (**Figure 7A**). Prolonged contact time (1 s) led to increased adhesion frequencies (75–90%), increased adhesion forces (from 300 to 2400 pN) and rupture lengths in the same range as at short contact time, yet with higher frequencies of short rupture distances (**Figure 7B**). At short contact time, most force distance curves presented multiple well-defined individual peaks of 50–100 pN (**Figure 7A**, right histogram inset). According to previous observations, we attribute those multiple peaks signatures with flat regions preceding each peak to type IV pili interaction with MB6 surfaces (Touhami et al., 2006; Biais et al., 2010). The absence of such structures on bacterial images (**Figures 4A,B**) suggest that TC5 pili are fragile, short or could be retracted during sample drying. On 1 s contact time force curves, similar peaks were sometimes observed but the short distances interaction at higher forces (>300 pN) seemed to govern the adhesion of cell on MB6 surfaces. Such first large force and short distance adhesive events phenomenon could be attributed to the outer membrane surface property itself that needs longer contact time for interaction rather than appendages or adhesives molecules that would lead to longer rupture lengths. Analysis of TC10 cells led to similar results, still with few differences. Increasing the contact time did not significantly increases the adhesion frequency or the range of forces. As for TC5, force curves signatures obtained for TC10 suggested type IV pili interaction and this conclusion was confirmed by AFM images (**Figure 4D**). For TC11 cells, the adhesion frequency did not increase with contact time. However, adhesion forces of some cells significantly increased up to 10 nN (**Figure 7F**). These high forces corresponded to short rupture distances peak suggesting strong hydrophobic interactions between the cell surface and the MB6 substrate. At short contact time, force curves frequently showed a first adhesive event with sometimes a sawtooth pattern (**Figure 7E**, inset in right histogram, upper curve). This first peak may correspond to cell surface proteins interacting with MB6 surface and strengthened in force and number under prolonged contact time. On short contact time curves, although no pili were detected on bacterial images (**Figures 4E,F**), peaks following the initial adhesive event presented signatures that could be attributed to pili and as for TC5, we hypothesize that those pili were fragile, short or could be retracted on image samples. Force curves obtained after prolonged contact time revealed sawtooth pattern with regular peaks and long rupture distances. Based on previous observations, these signatures could correspond to proteins interacting with the surface and containing multiple repeats that are unfolded upon bacterial pulling from the substrate (Alsteens et al., 2009; Beaussart et al., 2014; El-Kirat-Chatel et al., 2014b).

**FIGURE 8:**
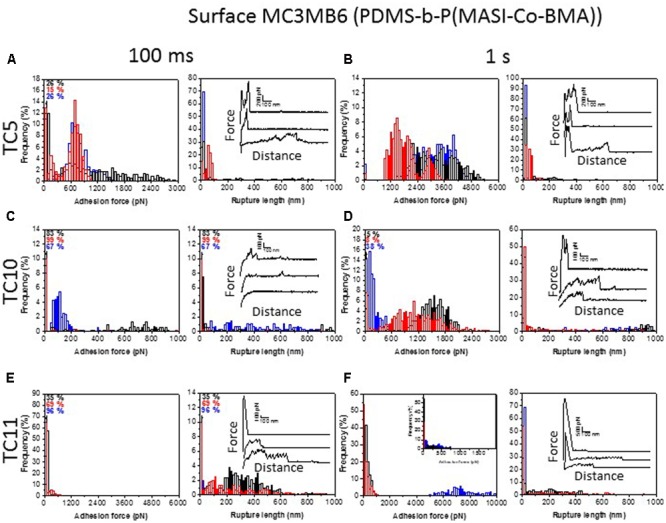
Single-cell force spectroscopy analysis on surface MC3MB6. Adhesion force (left) and rupture length histograms with representative retraction force profiles (right) obtained by recording multiple force-distance curves between single TC5 **(A,B)**, TC10 **(C,D)**, or TC11 **(E,F)** bacteria and surface MC3MB6 at short (100 ms, **A,C,E**) or prolonged (1 s, **B,D,F**) contact times. Black, red, and blue colors represent results from three cells from independent cultures (*n* > 400 force-distance curves for each cell).

The MC3MB6 surface was used similarly to evaluate the impact of the surface chemistry change on bacterial adhesion of TC5, TC10, and TC11 cells. **Figure 8** shows the adhesion force and rupture length histograms, together with representative force curves, obtained between TC5 (**Figures 8A,B**), TC10 (**Figures 8C,D**), and TC11 (**Figures 8E,F**) cells and surfaces MC3MB6 at short (**Figures 8A,C,E**) and prolonged (**Figures 8B,D,F**) contact times. TC5 cells presented high frequency adhesion toward surface MC3MB6 (more than 70% at short contact times and about 100% at prolonged contact times). Increasing contact time resulted in higher adhesion forces (from 300–2000 pN to 1200–4800 pN). Force curves recorded for TC5 on surface MC3MB6 presented large initial force peaks followed by smaller forces that may correspond to stretching of cell surface molecules. Compared to results obtained on surfaces MB6, force curves recorded on surface MC3MB6 rarely presented pili signature, suggesting that TC5 pili are mostly involved in interaction with surface MB6 and that the interaction with surface MC3MB6 is governed by the cell wall itself together with surface adhesive molecules. TC10 cells presented slightly similar adhesive profile on surface MC3MB6 and surface MB6. Its adhesion to both surfaces is lower in term of frequency and force than the adhesion of TC5. Based on force curves shape, this adhesion seems to be mainly controlled by pili at short contact time (small peaks at long distance and visualization of pili on image **Figure 4**) and at longer contact time, the cell adhesion through pili seems to be reinforced by cell wall and surface molecule (large initial peak and sawtooth pattern of molecules unfolding). TC11 cells were slightly more adhesive to surface MC3MB6 than what was observed for surfaces MB6. Still, the interaction looks similar with almost no pili signature but rather protein unfolding and large initial peaks suggesting that TC11 adhere mainly through cell wall hydrophobicity and adhesive macromolecules containing repeated domains unfolded upon pulling. Phenotypic heterogeneity in adhesion was also more obvious on MC3MB6 for TC11 than for the two other strains, whether on short or long contact time.

To validate the specificity of the measured adhesion forces and rule out the possibility of artifact associated with the cell probe preparation, a control experiment was performed using a 1 s contact time (Supplementary Figure S2). Use of polydopamine-coated probes instead of bacterial probes led to a major reduction of adhesion frequency and no signatures similar to what we observed for cells were present. This control indicates that the adhesive events measured above reflect the interactions between bacteria and coatings.

Taken together, these results show first that TC5 was the most adhesive of the three strains on both surfaces in terms of frequency and presented large adhesion forces, in particular on the hydrophobic surface, MC3MB6, while TC10 showed a weaker adhesion on both surfaces with adhesion forces averaging 1000 or 1200 pN. The influence of surface chemistry is mostly observed for TC5. This comes in contrast with the results found at the population level, as TC11 was the bacteria that adhered the most efficiently on polystyrene, glass as well as on MB6 and MC3MB6, even though adhesion on the antifouling surfaces was overall very low (**Figure 4**). Second, different extracellular components seem to be involved in the three strains adhesion on the surfaces. Short distance interactions at higher forces govern adhesion of TC5 and TC10 on the surfaces (Dufrene, 2015). Adhesion seems to be controlled by pili, cell wall on MB6 (and MC3MB6 for TC10) and by cell wall and stretching of surface molecules on MC3MB6. TC11 adheres mainly through cell wall hydrophobicity and adhesive macromolecules containing repeated domains unfolded upon pulling. Third, some TC11 cells were slightly more adhesive to both surfaces (with long rupture distances) than others. While TC5 and in a lesser extent TC10 showed a more homogenous response toward the surfaces, TC11 presented heterogeneous adhesion profiles toward both surfaces, with some bacterial cells presenting weak adhesion forces and some of them presenting very strong ones. Phenotypic heterogeneity within a population, which corresponds to the expression of substantial phenotypical differences by individuals when they are in a similar context, is thought to allow better chance of survival for the population as a whole entity. A subpopulation may be then better equipped to face stressful situations and settle in new environmental niches. This heterogeneity can come from variations of gene expression at the single cell level but also from allelic variations (Davis and Isberg, 2016). Some bacteria are more susceptible to genetic variations than others (for instance the ones undergoing phase variation). These phenomena have been described for instance in biofilm with the apparition of persisters as well as in relation with QS dependent mechanisms due to highly heterogeneous gene expression at a single cell level (Grote et al., 2015). This is most likely a widespread phenomenon, which just started to be highlighted in the literature with the development of single cell approaches, even though this variability may differs from a bacterium to another. This emerging evidence of phenotypical variability need to be studied more precisely at the molecular and cellular level in order to understand how these variations can make a subpopulation adapt and survive in an environmental niche. It is possible that TC11 fits to this description and can present at the population level a better fitness when facing a new environment than TC5 or TC10. Phenotypic heterogeneity, and differential bacterial-bacterial collaborative interactions involved in biofilm formation could explain the reason why, despite a weaker adhesion at the single cell level, TC11 is able to form more biofilm than TC5 including in ASW (data not shown). Further studies would be required using, for instance, other single cell techniques, such as the newly developed single cell RNA-seq, allowing a broader vision of the variability to confirm these results (Davis and Isberg, 2016).

## Author Contributions

SE-K-C has performed the AFM/SCFS experiments to measure adhesion forces between live bacteria and the surfaces under the supervision of YD. AP and PVO have performed the microbiology adhesion assays and CLSM experiments under the supervision of MM. TD has synthetized and characterized the copolymers under the supervision of CB and LB.

## Conflict of Interest Statement

The authors declare that the research was conducted in the absence of any commercial or financial relationships that could be construed as a potential conflict of interest.
