# Supplementary material for: Phenotypic Heterogeneity in Attachment of Marine Bacteria toward Antifouling Copolymers Unraveled by AFM
Source: Front Microbiol. 2017 Jul 27;8:1399. doi: 10.3389/fmicb.2017.01399 (PMC5529340; doi:10.3389/fmicb.2017.01399)
Supplement: Supplementary file 1 [file Data_Sheet_1.docx]

Supplementary Material

**Phenotypic heterogeneity in attachment of marine bacteria towards antifouling copolymers unraveled by AFM**

Sofiane El-Kirat-Chatel, Aurore Puymege, The Hy Duong, Perrine Van Overtvelt, Christine Bressy, Lénaïk Belec, Yves F. Dufrêne, Maëlle Molmeret*

*** Correspondence:** molmeret@univ-tln.fr

# Supplementary Data/Supporting Information

1.1. Materials

Tert-butyldimethylsilyl methacrylate (SiMA) was synthesized as described elsewhere (Nguyen et al., 2005), distilled under reduced pressure and stored under argon before use. α-hydroxyethylpropoxyl-ω-propyl poly(dimethylsiloxane)s (PDMS-OH) of 10,800 g mol-1 (10k PDMS-OH) was used as received. 4-cyano-4-(dodecylsulfanylthiocarbonyl)sulfanylpentanoic acid (CTA) (Strem Chemicals) was used as received. Dicyclohexylcarbodiimide (DCC), tert-butyl(chloro)dimethylsilane, 4-dimethylamino pyridine (DMAP), hexane, methanol, acetonitrile and ethyl acetate were purchased from Sigma-Aldrich and used as received. 2,2’-azobis(isobutyronitrile) (AIBN) was purchased from Sigma-Aldrich and purified by recrystallization from methanol. Butyl methacrylate (BMA), toluene and dichloromethane (DCM) were purchased from Sigma-Aldrich and distilled under reduced pressure to remove inhibitors before use.

1.2. Polymerization procedures

All RAFT polymerizations were carried out in distilled toluene, at 70°C, using AIBN as initiator, with a molar ratio [CTA]/[AIBN] = 5.

The CTA molar concentration dependent on the target number-average molar mass (Mn,target) was calculated with eqn. 1 considering a total monomer conversion:

M_(n,target)=(〖[M]〗_0×M_monomer×ρ)/〖[CTA]〗_0 +M_CTA (1)

Where [M]0 is the initial monomer concentration, Mmonomer is the molar mass of the monomer, ρ is the fractional conversion, [CTA]0 is the initial concentration of macro-RAFT agent, and MCTA is the molar mass of the macro-RAFT agent.

Monomer concentration and molar ratio of CTA or macro-CTA/AIBN and BMA/MASi were fixed to 1.5 M, 5 and 6, respectively. Polymerization procedure was described elsewhere (Duong et al., 2014).

1.2.1. Synthesis of P(SiMA-stat-BMA) copolymer (MB6)

10 mL of a solution composed of AIBN (0.0029 g, 0.017 mmol), CTA (0.018 g, 0.045 mmol), SiMA (0.4211 g, 2.1 mmol), BMA (1.8309 g, 12.9 mmol) in toluene were charged into a dried two-neck flask along with a magnetic stirrer bar. The solution was then deoxygenated by bubbling argon for 40 min at room temperature. The reaction flask was placed in an oil bath preheated to 70 °C. At the end of the reaction, a sample was taken with a degassed syringe to evaluate the monomer conversion by 1H-NMR comparing the signals of the reactive double bond (5.65 ppm for SiMA and 5.6 ppm for BMA) from monomers with the methyl protons –Si(CH3)2– from SiMA and PSiMA (from 0.34 to 0.15 ppm) and the methylene protons –O–CH2– from BMA and PBMA (from 4.2 to 4.0 ppm). Then, the reaction mixture was cooled to room temperature and precipitated in methanol. The obtained polymer was rinsed with methanol three times and dried under vacuum at 40 °C to a constant weight. The molar masses and dispersity values of purified random copolymers were determined by TD-SEC.

1.2.2. Synthesis of PDMS-block-P(SiMA-stat-BMA) (MC3MB6) copolymer

The macro-chain transfer agent (macro-RAFT agent) MC3 was synthesized by the esterification of monohydroxyl-terminated poly(dimethylsiloxane)s with a carboxylic acid end-functionalized trithiocarbonate RAFT agent catalyzed by DCC/DMAP (Duong et al., 2014). Then, MC3MB6 copolymer with a target Mn value of 62,000 g.mol-1 was synthesized using MC3 as macro-RAFT agent. The macro-RAFT agent concentration was estimated from Eqn. 1 for a given target Mn value of the diblock copolymer. Mn,target is defined at 100% of monomer conversion.

Number-average molar mass and dispersity values of purified MB6 and MC3MB6 copolymers were determined by TD-SEC. (Table 1).

# Supplementary Figures and Tables

**Supplementary Table S1. List of strains used in this study** (adapted from([Brian-Jaisson et al., 2014](#_ENREF_14)))

|  |  |  |  |  |
| --- | --- | --- | --- | --- |

| **Name** | **Taxonomic best alignement hit** | **Putative designation** | **Motility** | **Biofilm formation *** |
| --- | --- | --- | --- | --- |
| TC5 | *Polaribacter dokdonensis* strain DSW-5 | *Polaribacter* sp. TC5 | Non motile | None |
| TC8 | *Pseudoalteromonas lipolytica* strain K-W45 | *Pseudoalteromonas lipolytica.*TC8 | Motile | 24h** |
| TC9 | *Shewanella surugensis* strain c959 | *Shewanella* sp. TC9 | Motile | 24h |
| TC10 | *Shewanella japonica* strain KMM 3299 | *Shewanella* sp. TC10 | Motile | 48h |
| TC11 | *Shewanella pneumatophori* strain SCRC-2738 | *Shewanella* sp. TC11 | Motile | 24h |

* in marine broth (MB)

** biofilm formation observed from the indicated time


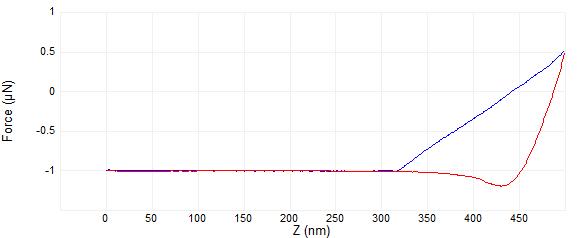

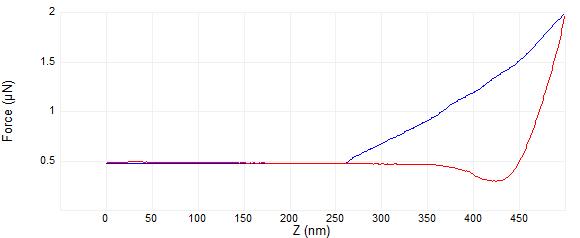


A

B

**Supplementary Figure S1. AFM force curves of A) MB6 and B) MC3MB6.**


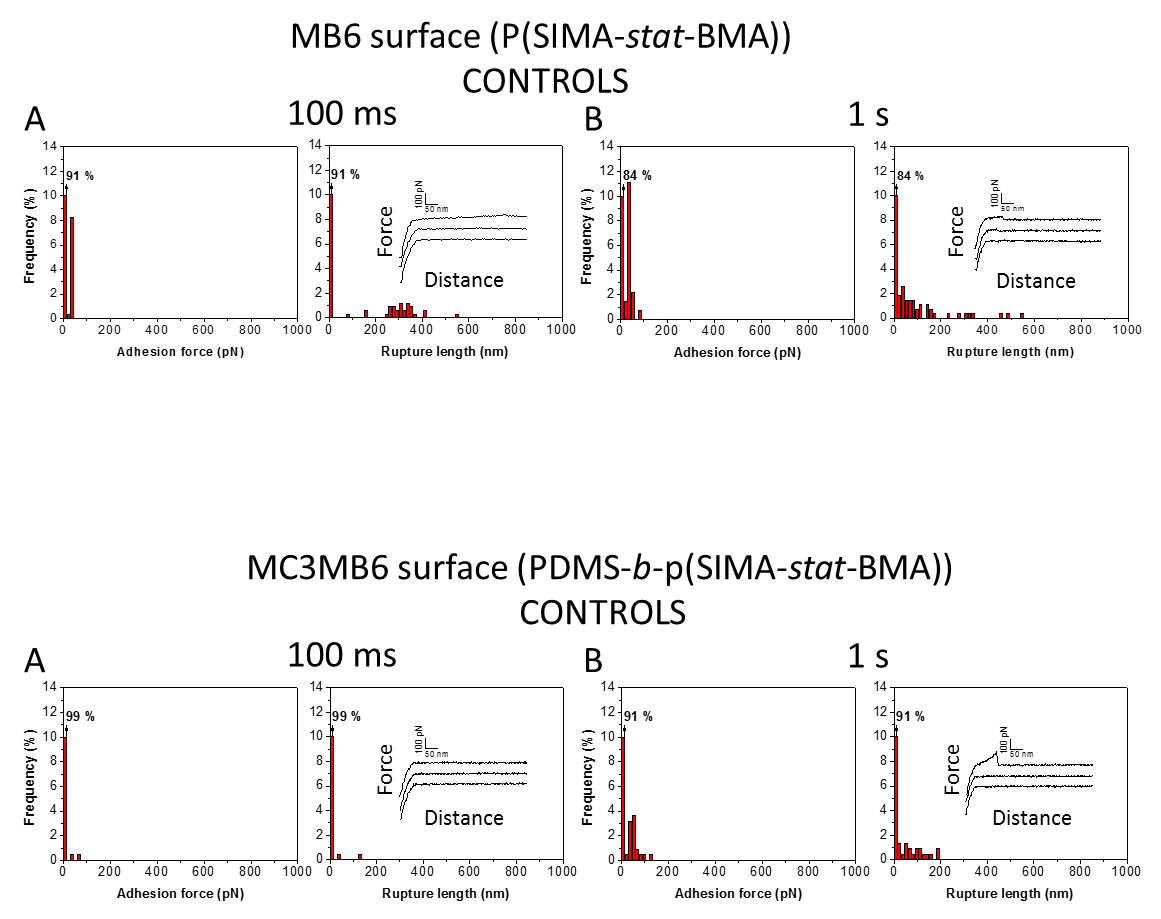


**Supplementary Figure S2.** **Single-cell force spectroscopy analysis using the silica microbeads as a control on the MB6 and MC3MB6 surfaces.** Adhesion force (left) and rupture length histograms with representative retraction force profiles (right) obtained by recording multiple force−distance curves between polydopamine coated colloidal probe and surface MB6 (A and B) or MC3MB6 (C and D) at short (100 ms, A, C and E) or prolonged (1s, B, D, F) contact times. (*n* > 400 force−distance curves).
